# Seed Dormancy and Pre-Harvest Sprouting in Rice—An Updated Overview

**DOI:** 10.3390/ijms222111804

**Published:** 2021-10-30

**Authors:** Soo-In Sohn, Subramani Pandian, Thamilarasan Senthil Kumar, Yedomon Ange Bovys Zoclanclounon, Pandiyan Muthuramalingam, Jayabalan Shilpha, Lakkakula Satish, Manikandan Ramesh

**Affiliations:** 1 Department of Agricultural Biotechnology, National Institute of Agricultural Sciences, Rural Development Administration, Jeonju 54874, Korea; pandiannsp7@gmail.com (S.P.); seninfobio@gmail.com (T.S.K.); angez9914@gmail.com (Y.A.B.Z.); 2 Department of Biotechnology, Sri Shakthi Institute of Engineering and Technology, Coimbatore 641062, India; pandianmuthuramalingam@gmail.com; 3 Department of Biotechnology, Alagappa University, Karaikudi 630003, India; mrbiotech.alu@gmail.com; 4 Department of Biotechnology, School of Life Sciences, Pondicherry University, Puducherry 605014, India; shilphajayabalan@gmail.com; 5 Department of Biotechnology Engineering, Ben-Gurion University of the Negev, Beer Sheva 84105, Israel; lsatish@post.bgu.ac.il

**Keywords:** abscisic acid, gibberellin, pre-harvest sprouting, growth hormones, rice, seed dormancy, QTLs, transcriptomics

## Abstract

Pre-harvest sprouting is a critical phenomenon involving the germination of seeds in the mother plant before harvest under relative humid conditions and reduced dormancy. As it results in reduced grain yield and quality, it is a common problem for the farmers who have cultivated the rice and wheat across the globe. Crop yields need to be steadily increased to improve the people’s ability to adapt to risks as the world’s population grows and natural disasters become more frequent. To improve the quality of grain and to avoid pre-harvest sprouting, a clear understanding of the crops should be known with the use of molecular omics approaches. Meanwhile, pre-harvest sprouting is a complicated phenomenon, especially in rice, and physiological, hormonal, and genetic changes should be monitored, which can be modified by high-throughput metabolic engineering techniques. The integration of these data allows the creation of tailored breeding lines suitable for various demands and regions, and it is crucial for increasing the crop yields and economic benefits. In this review, we have provided an overview of seed dormancy and its regulation, the major causes of pre-harvest sprouting, and also unraveled the novel avenues to battle pre-harvest sprouting in cereals with special reference to rice using genomics and transcriptomic approaches.

## 1. Introduction

The first crucial step in the life cycle of plants and the basis of agricultural production is seed germination. In contrast, seed dormancy is an important agricultural trait in cereal crops. Seed dormancy is an adaptive trait that allows seeds of many species to remain dormant until conditions are ideal for germination. On the other hand, seed dormancy is a complicated feature that is influenced by a variety of endogenous and external variables; it is often referred to as one of the most poorly understood aspects of seed biology [1]. This involves a complicated set of physiological and biochemical processes that are influenced by both intrinsic seed dormancy and a variety of extrinsic environmental cues. The plant hormones abscisic acid (ABA) and gibberellic acid (GA) are the major endogenous regulators that antagonistically control the seed dormancy and germination in several plant species [2,3]. The production of ethylene, nitric oxide, brassinosteroids, and reactions to light, temperature, and other external environmental factors all play a role in seed dormancy and germination [4]. Prolonged seed dormancy can lead to overgrown plants or weed problems in cultivated areas, while insufficient dormancy can result in premature germination. The germination of seeds inside the kernel while the panicle is still attached to the stem of the plant is a phenomenon known as pre-harvest sprouting [5]. It is a significant cause of quality and productivity loss in a variety of food crops such as *Zea mays*, *O. sativa*, and *T. aestivum*, especially in humid climates [6,7]. It also promotes starch hydrolysis in endosperm, resulting in lower grain weight and creating a favorable environment for saprophytic fungi [8]. In addition, selection for rapid, uniform germination over the course of domestication and breeding has reduced seed dormancy and thus increased the susceptibility of cereal plants to pre-harvest sprouting [9].

*O. sativa* L. is the primary and stable food source for more than half of the world’s population. Food security therefore requires the maintaining of its production in the face of global warming. In rice, ABA regulates seed dormancy mainly through the balance of ABA/GA ratio [10]. The major key players of the ABA signaling pathway have been analyzed in various plants: *TaMFT* and *TaPHS1* in wheat [11]; *ABI3*, *DOG1*, and *LEC2* in *Arabidopsis* [12]; *VP1* in maize [13]; *Sdr4*, *OsDSG1*, *OsAB13*, *OsAB15*, *PHS8*, *PHS9*, *OsNCED3*, *OsVP1*, *OsPDS*, *β-OsLCY*, *OsFbx352*, *OsMFT2*, *OsZDS*, and *OsCRTISO* in rice [3]. These players associated with seed dormancy and germination are linked to the biosynthesis, perception, catabolism, and signal transduction of ABA, revealing their crucial roles in the control of seed dormancy [14]. Key enzymes responsible for catalyzing the cascading reactions in the biosynthesis of GA, such as GA 3-oxidase (*GA3ox*) and GA 20-oxidase (*GA20ox*), have been identified in rice (*O. sativa* L.), wheat (*T. aestivum)*, *Arabidopsis*, and barley (*Hordeum vulgare)* [15,16]. However, the exact regulatory mechanisms of these players remain elusive. Environmental changes can also have a significant impact on rice development and yield. For instance, prolonged rain and high humidity during rice maturation can cause seed germination in rice panicles prior to harvest, resulting in significant economic and productivity losses [17,18]. Due to the long period of rainy weather in early summer and autumn in Southeast Asia, pre-harvest sprouting is widespread in rice [7].

Significant improvements in next-generation sequencing (NGS) have paved the way for a promising generation of diverse omics approaches such as genomics, transcriptomics, proteomics, metabolomics, ionomics, hormonomics, and phenomics, which have also been well implemented in crops, especially rice [19,20]. These omics-based approaches, particularly transcriptomics with high-throughput techniques, will enable molecular analysis of the precise systems regulating seed dormancy in rice and other crops. As a result, comprehensive molecular processes of all components upstream or downstream of ABA and GA signaling pathways in rice will need to be explored in the future using a blend of genomic and genetic techniques. Keeping these lacunae in mind, in this review, we aimed to provide an overview of seed dormancy and the role of hormones in the pre-harvest sprouting in rice. Here, we also discuss the genes and quantitative trait loci (QTLs) and the functional genomics of pre-harvest sprouting resistance genes with a focus on rice.

## 2. Seed Dormancy and Germination—A Game of Hormones

**Figure 1:**
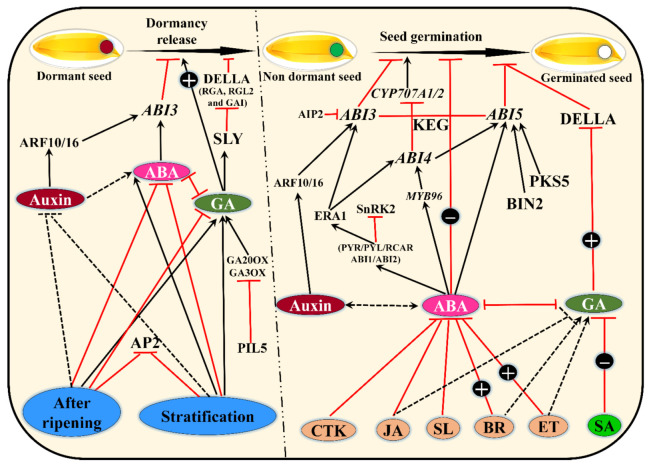
Regulatory phytohormone networks in seed dormancy and seed germination. Three major phytohormones, including auxin, abscisic acid (ABA), and gibberellin (GA), are key players in seed dormancy and germination. Mature seeds are dormant and contain a high level of ABA and a low level of GA. Several transcription factors (ABI4, DDF1, OsAP2-39, AP2, and CHO1) are involved in the seed dormancy stage by positively regulating (+) the accumulation of ABA and decreasing the GA content. While seed dormancy is broken, the seed becomes nondormant and the initiation of germination can start. At this stage, the ABA/GA balance is kept by positive and negative regulation signals of almost all other phytohormones, including ethylene (ET), brassinosteroids (BRs), jasmonic acid (JA), salicylic acid (SA), cytokinins (CTKs), and strigolactones (SLs). Here, transcription factors including ARFs, MYB96, ABI3, ABI4, and ABI5 regulate ABA biosynthesis by interacting with *CYP707A1* and *CYP707A2*, while GA-negative regulation (-) is ensured by *DELLA* genes. The balance is constantly maintained until the seed emergence step.

In the metabolism of plants, seed dormancy and germination are two separate biochemical and physiological processes [21]. The principal physiological factors that determine the dormancy and germination of seeds are the plant hormones, predominantly ABA and GA [22]. ABA affects dormancy formation and persistence favorably, whereas GA increases germination. GA promotes germination by initiating embryo activity, overcoming the mechanical restraints imposed by the aleurone or testa, and stimulating the growth of the embryo [3]. Generally, in plants, the biologically active GA level is maintained by a balance between degradation and biosynthesis [15]. The regulatory mechanisms controlling dormancy mitigation and seed germination are underpinned by an intricate balance in ABA and GA metabolism and their antagonistic hormonal interactions, in which reactive oxygen species and Ca^2+^-dependent signals serve as signal progenitors, integrators, or transducers. The ABA/GA ratio also regulates the status of dormancy in plants, whereas other hormones (e.g., Jasmonates) are known to impact seed dormancy predominantly through their effects on the ABA/GA ratio (Figure 1). During the dormancy, endogenous/exogenous ABA levels are controlled by fine-tuning hormone production through disintegration of carotenoid precursors and silencing by 80-hydroxylation in distinct seed tissues [23]. After seedling establishment, ABA slows and increases sprouting over time by inducing an adaptive characteristic known as primary dormancy throughout seed maturity. The time period of primary dormancy is significantly influenced by environmental variables throughout seed development, particularly drought [24]. ABA breakdown anticipates the triggering of seed germination besides GA following seed shedding, allowing dormancy to be liberated. Evidence suggests that the ABA/GA ratio integrates environmental cues such as daylight, temperatures, and ammonia—nitrogen, and works against embryo development and endosperm thinning [25]. Soaking the *O. sativa* seeds with GA resulted in breaking the seed dormancy [26], while the application of paclobutrazol (an antagonist of GA) delayed dormancy in *Sorghum bicolor* [27].

PYR-like/regulatory PYR-like/ABA receptor components are found in seeds and vegetative organs, and they internalize and regulate protein phosphatase 2C when ABA is present. This multigene family is involved in ABA sensing both in seeds and vegetative tissues. As protein phosphatase 2C is inactive, this permits SNF1-related kinase-2 to become activated, which then causes SNF1-related response elements to bind to their promoter regions [28]. DELAY OF GERMINATION-1 (DOG-1) is a master regulator of primary dormancy that acts in concert with ABA to delay germination [29]. In order for DOG-1 to maintain primary dormancy, it needs protein phosphatase 2C, which is provided by ABA. DOG-1 boosts ABA signaling through interacting with the protein phosphatase 2C ABA HYPERSENSITIVE GERMINATION, where DOG-1 (by using DOG1–heme complex) inhibits its activity to elevate ABA sensitivity and imposes primary dormancy. Heat stress during grain filling had almost no effect on *OsDOG1-like* gene expression in imbibed embryos, but the genes *OsNCED2* and *OsABA8′OH3* play the most important roles in ABA biosynthesis [30,31]. During early grain filling, the resistant cultivars slow the seed germination and are independent of primary dormancy release, though susceptible cultivars generate greater grain chalkiness when subjected to heat stress [32]. DNA methylation of ABA catabolism-related and alpha amylase gene promoters inhibits the germination of heat-stressed embryos in plants toward abiotic stress, notably during the grain filling process [31]. The functions of two new genes, viz. *ABA-DEFICIENT-4* and *NEOXANTHIN-DEFICIENT-1*, are uncharacterized and precludes neoxanthin production, which is essential for ABA accumulation [33].

According to hormone balance theory, as ABA signaling drops significantly within a week of seed maturation, GA signaling increases, which finally leads to seed germination [34]. Previous dormancy research in model crops and cereals have shown a clear link among both ABA and GA signaling and seed dormancy and dormancy loss, laying the groundwork for dormancy control in several other crops. Previous studies have importantly pointed out that: (i) during dormancy breakdown, ABA levels and/or sensitivity decrease, whereas susceptibility to GA increases; (ii) GA-insensitivity is linked to a lack of germination percentage in dormant seeds at seedling emergence, while GA stimulates germination in nondormant seeds; (iii) dormancy is abandoned in cycles when dormant seeds after-ripen, as shown by variations in sensitivity to ABA and/or GA [35,36]. For the gene that controls transcription in the aleurone zones of *H. vulgare*, *O. sativa*, *T. aestivum*, as well as other cereals, *GAMYB* promotes alpha amylase gene expression in a GA-dependent approach [37]. Dormancy is sustained and rigorously modulated by hormonal signaling networks that are controlled. Few investigations have proved that increased GA production and hormone biosynthesis caused by *GA_20_-Oxidase* gene expression level lower ABA sensitivity and enhance GA sensitivity after ripening, whereas as dormancy decreases, the expression of GA2-oxidase, a GA catabolism gene, tends to decrease. In addition, with after-ripening, the GA-INSENSITIVE DWARF-1 hormone receptor increases, and when dormancy is lost, ABA hormone accumulation diminishes due to increased ABA catabolism [36]. Additional hormones, also including jasmonic acid isoleucine, brassinosteroid, and indole-3-acetic acid (IAA), have lately been involved in seed dormancy and consequently germination or premature germination on the spike [38].

During seed maturation, plant hormones positively regulate reserve accumulation, inhibit embryo growth, and induce the desiccation tolerance and primary dormancy. Over the last few decades, significant efforts have been undertaken to comprehend the plant hormone communication pathways that govern dormancy and germination. Hormone signaling pathways governing dormancy and germination have been intensively studied only over the last few years. Thus, it is clear that additional research is needed to learn about hormone transport and communication, as well as biophysical and mechanical tissue characteristics, which all point to the importance of tissue-specific control and the interaction of signals during this critical stage of seed dormancy and germination. Recent studies in cereal genomics have opened the floodgates to unravel the key players involved in ABA and GA signal transduction pathways and metabolism. Further, studies on ABA-GA cross-talks in cereal crops have been one of the novel avenues to dissect the molecular dynamisms in the functionalities of seed germination and dormancy [3,39,40,41].

## 3. Pre-Harvest Sprouting

Cereals are the most important crops around the globe with an annual production over 2788 million tons [42]. Thus, cereals and their production are major bottlenecks by the large spectrum of environmental stressors, including the phenomenon of germination of grains, soon after maturation in the mother plant under wet/humid conditions, referred to as pre-harvest sprouting. It is often associated with severe yield losses and poor quality of grains in a wide range of cereals such as rice, wheat, maize, barley, rye, and oats [41,43,44,45]. Pre-harvest sprouting is a massive worldwide agricultural issue that has now been documented in Australia, Canada, China, Europe, Japan, South Africa, and the United States where it causes an annual economic penalty of one billion dollars on a global scale [46]. It occurs when the temperature and moisture levels are suitable for sprouting. Energy is produced in the grain by the breakdown of the primary store components, starch and protein, allowing the shoot to expand if the external environmental parameters for sprouting are met [18,47]. In addition to that, pre-harvest sprouting is mainly directed by both genetic and environmental factors, as well as interactions between these mentioned factors. High-pre-harvest-sprouting-resistance breeding varieties have significant implications for minimizing the yield loss and enhancing grain quality. The ever-increasing advancements in the genomic repositories/databases of rice and other crops integrated with transcriptomics and proteomics technologies have broadened our perspective for unravelling the physiological and functional regulatory mechanism of pre-harvest sprouting resistance at both transcriptomics and post-transcriptomics levels [45]. Further, pre-harvest sprouting resistance is associated with diverse physiological, developmental, and morphological features of grains on the spike, including pericarp color transparency, seed dormancy, permeability, enzymatic activity such as α-amylase, and hormone levels such as ethylene, ABA, and GA, all of which play a role during this process [48]. Several other factors, including hairiness, waxiness, and germination inhibitory compounds, enveloping the grains and ear morphology have also been associated with resistance to pre-harvest sprouting [49]. Among these, seed dormancy is the significant factor that controls the resistance. Therefore, alternative strategies and much attention have been subjected to deciphering the molecular cross-talks of seed dormancy as a means to enhance resistance in rice and other cereals through breeding programs.

## 4. Factors Affecting Seed Dormancy and Pre-Harvest Sprouting

Agricultural production must be progressively raised to improve human adaptation to hazards as the global population grows and natural disasters become more common. Even though the shorter dormancy period is believed to have enhanced the commercial productivity of cereals such as *O. sativa*, *H. vulgare*, *T. aestivum*, and *Z. mays*, the rapid germination percentage has led to pre-harvest sprouting in places with more rainfall, leading to economic consequences [46]. Pre-harvest sprouting, which occurs when embryos with less or no dormancy are exposed to external variables (a rain event) before harvest and germination on the spikelets, is an important evolving problem that impacts the end-use quality among several cereals [34,50]. One of most common methods of describing pre-harvest sprouting in model species and crops is the disruption of primary dormancy. In many crops, the absence of dormancy has resulted in lower productivity because seeds germinate too early before harvest, revealing a major knowledge gap in the control of seed dormancy.

Environmental factors such as light, temperature, and hydration along with physiological and biochemical characteristics influence not only the severity of primary dormancy, but also the features of secondary dormancy and the time necessary for effective dormancy emergence [51]. While certain plant species with dormant seeds have received the most recognition, hundreds of others have no seed dormancy and sprout viviparously mostly on the mother plant or soon after distribution. Plant physiology has been linked to the prevalence of recalcitrance and vivipary in a variety of plant species. Such plants’ biochemical and physiological properties are essential controls of seed physiology, which coordinate behaviors of the seedling and maturing plant to respective environmental conditions [52]. Desiccation-intolerant embryos have developed several times in different genera and are most prevalent in species that live in damp or flooded habitats. Natural selection in wetlands may not be able to remove certain seed varieties, or it may choose for abnormalities in hormone physiology that impact both maternal and embryonic cells simultaneously. The ratio of GA:ABA within the seeds is intimately connected to the influence of light on seed dormancy and germination. *A. thaliana* seed germination is selectively regulated by photoreceptor phytochromes in red or far-red light, while *H. vulgare* seed germination is inhibited by photoreceptor cryptochromes in blue light [53]. Blue light transduced over the cryptochrome blue light receptor can increase dormancy in *H. vulgare* by inducing the ABA-synthesizing enzyme *9-cis-epoxycarotenoid dioxygenase* and decreasing the expression of the ABA-catabolizing enzyme *8′-hydroxylase*, while red/far-red light showed no effect [54].

Using suitable procedures to prepare seeds for harsh circumstances is thought to be a good approach to decrease the negative impacts of environmental stressors on the plant while also increasing output. The seed priming methodology is one of the best approaches that has gained a lot of attention lately. Researchers have looked at the use of priming as a way to increase germination and seedling establishment in plants, including *Z. mays*, *O. sativa*, *T. aestivum*, and *Stevia rebaudiana* [55]. Plant seeds can sense environmental factors, viz. temperature, oxygen, and light, in both space and time [56]. *A. thaliana* accessions grown in cold environments tend to start *DOG-1* expression prior to seed maturation. *DOG-1* is implicated in the development of primary dormancy in the planting material in response to the cold seed-maturation temperatures. As a result, *DOG-1* is expected to be sensitive to the environment [57]. *DOG-1* gene research is complicated by the fact that it influences flowering and drought tolerance. *DOG-1* and primary dormancy status are both elevated after exposure to cold stress in maternal plants during seed development [58]. Seed dormancy and climate-dependent germination require competence to control germination time in natural environments. However, the level of dormancy cycling for many species in the field is not quantitatively related to environmental, physiological, and biochemical characteristics.

Low temperature raises grain susceptibility to pre-harvest sprouting via a number of regulators, with 10 °C causing a significant increase in the expression of *DOG-1*, which may also boost *GA2ox6* expression in Arabidopsis seed development. *OsSdr4* controls seed dormancy in *O. sativa* via the *OsDOG1L-1* pathway [59], which was revealed very recently in *A. thaliana* [60]. Cold temperatures have been shown to affect the quantity of phytohormones in *O. sativa* seeds. Low temperature (15 °C) upregulated the *OsGA2ox2*, *OsGA2ox5*, and *OsNCED2* genes through a reduction in the GA:ABA ratio, resulting in a lower germination percentage [61]. Cold stratification (4 °C) of *T. aestivum* has upregulated the jasmonate genes of *TaAOS* and *TaAOC*, which block the expression of the ABA-synthesizing genes *TaNCED1* and *TaNCED2* and increase seed germination [62]. Cold stress causes jasmonate synthesis, and methyl jasmonate stimulates dormancy breaking in dormant seeds, whereas methyl jasmonate prevents germination in after-ripened seeds. Differential temperatures interrupt the seed dormancy more efficiently than constant cold stratification for equal time intervals. This suggests that changing temperatures are instructional for plant growth, and that plants prefer to adapt to the temperature to enhance their tolerance and break the dormancy [63].

Physical characteristics including seed coat color, awn presence or absence, and epicuticle waxes have consistently been associated with variations in pre-harvest sprouting frequency [38]. In addition, various biochemical characteristics influenced during germination cause poor product qualities of cereal crops. Pre-harvest glyphosate treatment may have an influence on the biochemical and nutritional features of wheat bran and proteins, perhaps by disrupting biochemical processes essential for starch and protein stacking, resulting in variations in seed quality attributes [64]. Even though the molecular foundation for the physiological impacts is recognized, the genes associated with pre-harvest sprouting are less understood. Several studies have revealed that QTLs and various biochemical systems implicate a complex series of genes. The causative genes are frequently overlooked [38,65]. Some variables that alter dormancy and pre-harvest sprouting via signal transduction or amino acid activity have been discovered. The enzyme alanine transaminase, which interconverts glutamate to alanine, has been reported to enhance dormancy in *H. vulgare*, although the pathway is unknown [66]. The efficiency of amino acids in both *H. vulgare* and *T. aestivum* was reduced by a bifunctional α-amylase/subtilisin inhibitor from *H. vulgare*. While *T. aestivum* and *Secale cereale* were discovered to have genes that were similar to those found in *H. vulgare*, none of the cultivars examined resembled the same substantial decrease in activity [67].

## 5. Pre-Harvest Sprouting in Rice

Due to the excessive rainfall during grain maturation, pre-harvest sprouting is widespread in rice, especially in southwest Asian countries. In addition, the inhibitory effect of eugenol on hybrid rice seed germination and pre-harvest sprouting due to a significant reduction in α-amylase activity has been reported recently [26]. The frequency of incidence of pre-harvest sprouting has been known to increase primarily after the yellow-ripe stage of grain filling, which is thought to be influenced by the steady reduction in ABA content from its peak point at a given point during grain development until maturation. Further, the likelihood of sprouting is increased after heading once a certain temperature has been reached [68,69]. Rice develops the potential to sprout when it reaches the late grain filling stage, that is, after a certain amount of time has passed since grain filling was completed. In spite of inter-cultivar variations, this period in time occurs when above 50% of rice grains can sprout, roughly 35–45 days after heading and following the attainment of an accumulated temperature of 800–900 °C [70]. Furthermore, ABA content, which is associated with pre-harvest sprouting resistance and plays a role in seed dormancy, peaks about 5–15 days after heading and subsequently declines as grain filling progresses. In addition, upon high-temperature grain filling, granule-bound starch synthase activity diminishes, resulting in milled grain with low amylose content, high free-sugar content, and low starch crystallinity with rapid water absorption, all of which are possible causes for increasing the rate of pre-harvest sprouting [70]. It has a wide variety of negative implications, from instant loss of seed viability upon desiccation to a significant reduction in seed lifetime when embryo growth has not progressed that much. Pre-harvest sprouting initiates the synthesis of enzymes that increase reserve mobilization, resulting in significant changes in grain quality [8,51].

In rice and wheat crops, a link between dormancy and pericarp color has been established, with red-grained varieties showing increased resistance to pre-harvest sprouting. Two loci that affect the red-colored grain in rice have been found through genetic studies, one of which encodes a basic helix–loop–helix transcription factor that causes enhanced dormancy when introduced into white-grained rice [7,71]. A pleiotropic gene that affects ABA and flavonoid production in early seed development is shown to influence seed coat-induced dormancy, which is linked to pericarp color in lower epidermal cells [72]. In addition to organic substances, seed coat impermeability to water and/or oxygen is emphasized in the research of seed dormancy [73]. The increased frequency of severe weather events such as torrential rainstorms and typhoons has raised the risk of pre-harvest sprouting in rice [17,74], prompting further research into pre-harvest sprouting mechanisms, QTLs, and key regulatory genes with the aim of developing rice cultivars with improved resistance [45,50,59,71,75]. The QTL/key players are essential for gene pyramiding in breeding programs. Still, the functional and regulatory mechanisms are far from clear, which is why the progress in developing rice and other cereals pre-harvest sprouting resistance is inadequate.

## 6. Omics Approaches for Pre-Harvest Sprouting in Rice

**Figure 2:**
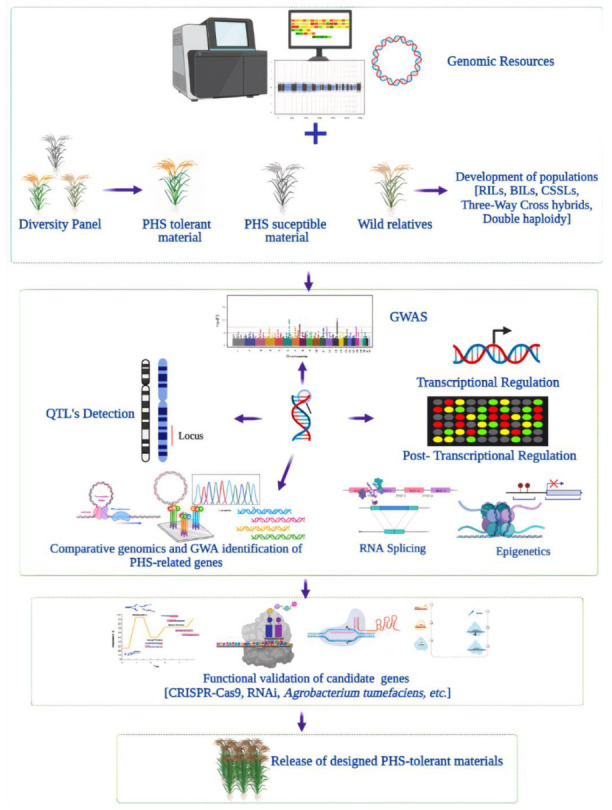
Omics-based approaches for the production of pre-harvest sprouting-resistant rice varieties. Applications of genomic selection (GS) such as estimating breeding values, developing improved models for prediction of parent and variety selection, as well as using genome-wide association studies (GWASs), marker-assisted selection (MAS), QTL studies, recombinant inbred lines (RILs), backcross inbred lines (BILs), chromosome segment substation lines (CSSLs), three-way cross hybridization, and double haploidy (DH) toward accelerated breeding.

Genomics-assisted breeding is one of the promising approaches to overcome pre-harvest sprouting and raise the yield potential to the level required to meet the fast-increasing global demand. During the last two decades, tremendous advances have been carried out with the identification of useful resistance/dominance genes. In Figure 2, we illustrate an overview of diverse genomics-assisted breeding approaches for effectively exploiting genomics research for pre-harvest sprouting resistance detection. The first and foremost stage in this process relied on the characterization of germplasm for the identification of promising genetics resources. The breeding program depends on the persistent phenotypic selection of resistant and susceptible parents to generate improved populations for further breeding processes. The genetic resources include contrasting materials derived from *O. sativa* subsp. *japonica* and *indica* varieties. In addition, wild relatives including *O. rufipogon* [76] and *O. nivara* [77] and weedy rice [78] were also employed. However, wild crop relatives’ usage for crop improvement remains a big challenge mainly due to hybridization barriers [79]. The genomics approach appears as a promising strategy, specifically for complex traits as it is less expensive and time-efficient [80]. The researchers are moving forward to the application of genomic selection such as estimating breeding values, developing improved models for prediction of parent and variety selection, as well as using various genetic and genomic approaches toward accelerated breeding [81]. The studies of the earlier detection of pre-harvest sprouting-related QTLs relied mostly on RFLP and SSRs markers. The recent progress of genetic studies based on molecular markers in pre-harvest sprouting, seed dormancy, low-temperature germination (LTG), and germination index (GI) is summarized in Table 1. Thus far, a total of 185 QTLs have been detected within all the 12 chromosomes of the rice genome (Table 1). Although MAS is an effective tool in modern plant breeding, it has been limited to simple traits with monogenic or polygenic inheritance in crops such as rice [82]. At an early stage of pre-harvest sprouting gene discovery, comparative genomics was also tested. The identification of orthologous genes by comparison with well-characterized pre-harvest sprouting and seed dormancy genes in barley and wheat revealed the presence of the hormonal *GA20-oxidase*-encoding gene [8].

**Figure 3:**
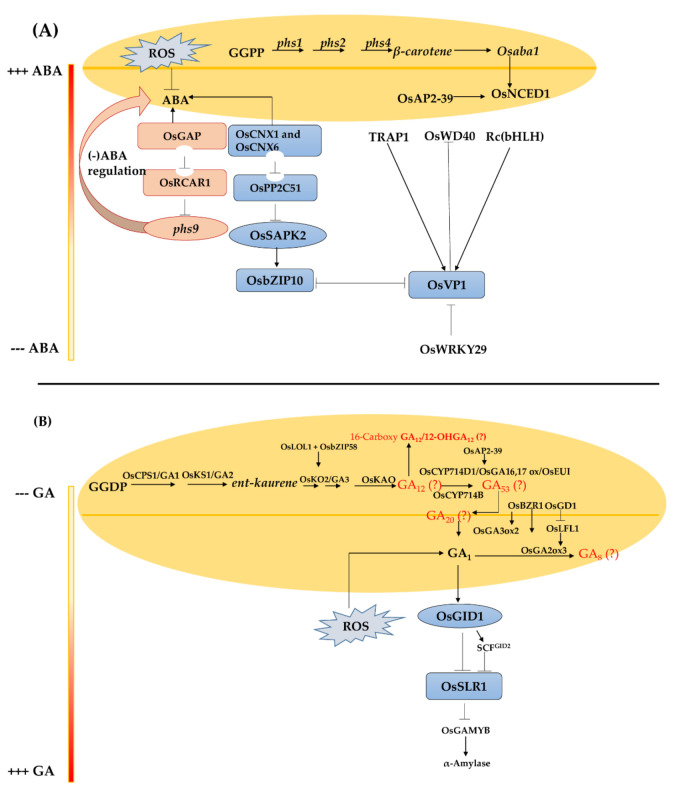
Regulatory networks of abscisic acid (ABA) and gibberellic acid (GA) of pre-harvest sprouting (PHS) in rice. (**A**) High accumulation of ABA in seeds can reduce the risk of PHS advent. At an early stage of ABA biosynthesis, beta carotenoid acts as a precursor with the conversion of geranylgeranyl pyrophosphate (GGPP). ABA signaling is regulated by downstream transcription factors such as OsbZIP, OsWRKY29, Rc(bHLH), OsWD40, TRAP1, and OsAP2-39. (**B**) Regulatory networks of gibberellin (GA) regulation of PHS in rice. Increased content of GA in seeds induce PHS. At an early step of GA biosynthesis, CPS-like, KS-like, and KO-like genes convert geranylgeranyl diphosphate (GGDP) to intermediate GA. The main core of GA synthesis in rice is defined by the complex GA_1_—OsGID1-OsSLR1-OsGAMYB. The red color of GA was predicted in *Arabidopsis thaliana* [123] (+++ is high hormone concentration whereas --- is low hormone concentration of both ABA and GA respectively).

Transcriptomics studies are considered an effective method for comparative transcriptome profiling, providing insight into the mechanism of gene regulation and networks controlling various complex biological processes, especially signal transduction [86]. High-throughput methods have been used to accurately characterize and quantify the complete set of RNAs in a given organ such as panicle, embryo and endosperm, tissues, or cells in different rice materials [114,115,116,117]. To determine the genes involved in pre-harvest sprouting and the corresponding biological processes such as seed dormancy, germination, and maturation, RNA and small-RNA sequencing were performed with different genotypes (Table 2). Candidate genes regulating hormones such as ABA (Figure 3A), GA (Figure 3B), and IAA were highlighted. These include transcription factors such as DREB (dehydration-responsive element-binding protein), basic helix–loop–helix transcription factor (bHLH), late embryogenesis abundant protein (LEA), NAC transcription factor, and CCAAT-HAP3 transcription factor and AP2-EREBP, highlighting the contribution of transcription factors as among the major players in mediating hormonal expression. It is well known that microRNAs (miRNAs) are dependent on hormonal regulation in plants [118]. Recently, Park et al. [117] identified two candidate miRNAs (osa-miR5827 and osa-miR1862h) associated with two pre-harvest sprouting-related genes *OsFbox594* and *OsbHLH084*, respectively. In addition to transcription factors, NCED (*OsNCEDs*) and CYP (*OsCYP707As*) genes were differentially expressed by comparing the transcriptome profiles of Korean cultivars [119].

High-quality genome resources enabled the detection of pre-harvest sprouting-related genes via GWASs and genome-wide identification approaches. Zhu et al. [121] pinpointed the role of the bZIP transcription factor *OsbZIP09*, whose expression is induced by ABA. The mutation of this gene inhibited pre-harvest sprouting in rice. By mining GWASs and transcriptome data, Shi et al. [122] found a significant effect of the variation in SNPs in the promoter region of the *Os9BGlu33* gene regarding germination index. In the same vein, taking advantage of a worldwide rice subpopulation, including *japonica* and *indica* populations, Magwa et al. [113] investigated candidate genes relative to seed dormancy by genome association analyses. A total of 54 loci were identified from which strong associations were mentioned with already cloned GA/IAA inactivation genes, including *GH3*-*2*, *GA2ox3*, and *EUI1*. Interestingly, one locus was found near the well-known pre-harvest sprouting resistance gene *Sdr4*. Using the GWAS approach on a 277-*japonica* rice panel, Lee et al. [70] pointed out ten candidate loci responsible for pre-harvest sprouting resistance. The candidate loci were predicted to be involved in ABA, GA, and IAA signaling pathways. 

From those large genetic and genomic resources, few candidate players have been functionally validated. Sugimoto et al. [59] identified the *Sdr4* gene as responsible for seed dormancy control. Interestingly, *OsVP1* exhibited a regulatory effect on the *Sdr4* gene via the ABA signaling pathway [3]. Transcription factors also play a crucial role for regulating the signal transduction and hormonal expression in rice. Hobo et al. [124], Wang et al. [9], and Wu et al. [125] demonstrated the interaction between *VP1* and *TRAP1* (bZIP transcription factor) and *Rc* (basic helix–loop–helix (bHLH) transcription factor) genes for ABA regulation. In addition, Xu et al. [62] demonstrated the implication of the glutaredoxin-mediated gene *PHS9* acting as a negative regulator of both ABA and ROS signaling during seed germination. The authors suggested a combinatory action of *PHS9* with *OsGAP* for reducing ABA signaling via the interaction with ABA receptor *OsRCAR1* (detailed in Figure 3A). The AP2 TF *OsAP2-39* was also functionally validated through an RNAi approach as a regulator of ABA and GA genes (*OsNCED1 and OsEU*) during seed dormancy [126].

## 7. Conclusions and Future Perspectives

In conclusion, ABA and GA act as hubs linking internal and external signals and antagonistically regulating pre-harvest sprouting. Advancements in our knowledge of the molecular mechanisms linked to dormancy, as well as quantitative genetics-based techniques, will enable new approaches for introducing the necessary level of dormancy into rice. Except in rice, mutant libraries that focus on seed dormancy and germination in few crop species have been developed [43]. Thus, creating mutant libraries in rice will be critical for future research. Furthermore, mutant analysis, as well as map-based cloning of the important gene loci, might bring new information about the seed dormancy in rice. Thus, genetic improvement for pre-harvest sprouting resistance requires a degree of primary seed dormancy recovery that is neither too strong nor too weak [5,9].

Considering the importance of environmental effects on seed dormancy and germination, we suggest a comparative investigation of the epigenome of pre-harvest sprouting-resistant and susceptible rice cultivars. In fact, increasing evidence has been in favor of DNA and histone methylation in regard to the pre-harvest sprouting resistance genetic mechanism [127,128]. The role of the ARGONAUTE4_9, a DNA methylation RNA-dependent gene, has been proved in the wheat pre-harvest sprouting resistance mechanism. However, the epigenetic framework of pre-harvest sprouting in rice is still elusive. Therefore, deciphering the epigenetic factors contributing to the pre-harvest sprouting resistance regulation in rice will lay a foundation for a deep understanding of the full machinery in real environmental cues. Moreover, an intensive validation of the existing candidate genes should be processed via CRISPR-Cas9 [129], RNAi [130], and super-*Agrobacterium tumefaciens*-mediated transformation [131] as a notable example. Altogether, post-transcriptional regulation encompassing, splicing RNA, and epigenetics offer novel avenues for unravelling the mechanism of resistance of pre-harvest sprouting in rice. Ultimately, a deeper comprehension of the whole machinery will provide a gain for designing agronomically improved rice. Further, molecular breeding programs will allow the exploitation of molecular markers in the screening of rice germplasm for pre-harvest sprouting resistance and aid in the development of pre-harvest sprouting-resistant rice varieties.

## Figures and Tables

**Table 1:** Summary of quantitative trait loci and genome-wide association studies for pre-harvest sprouting-related traits identified in rice.

Specialization of Study	Traits	QTL/Genes	Growth Condition	Markers Type	Markers No.	Mapping Population	Parents	References
QTL	PHS	qPHS-1-1, qPHS-1-2, qPHS-4, qPHS-5, qPHS-7, qPHS-8	NG, Yellow and White paper bag	RFLPs	6	71 F6 RILs	IR64 (*Indica*) × Asominori (*Japonica*)	[83]
	PHS	qPHS1-1FC, qPHS1-2FC, qPHS1-1GC, qPHS1-2GC	FC, GH	SNPs	8	88 F2:3 RILs	Jinsang (*Japonica*) × Gopum (*Japonica*)	[84]
	SD	qSD1-1, qSD1-2						
	LTG	qLTG1-1, qLTG1-2						
	SD	qSDR9.1 and qSDR9.2	FC	SNPs	2	44 BC4F5 CSSLs	Owarihatamochi (*Japonica*) × Koshihikari (*Japonica*)	[85]
	PHS	qPHS-3FD, qPHS-11FD, qPHS-3GH, qPHS-4GH, qPHS-11GH	FC, GH	KASP, CAPS, SNPs	5	F9 160 RILs	Odae (*Japonica*) × Unbong40 (*Japonica*)	[86]
	PHS	RM4108-RM5849, RM3455-RM6905	FC	SSRs, SNPs	2	79 N-BILs, 48 BC3F1 N-CSSLs, 41 BC4F1 K-CSSLs	Nipponbare × Koshihikari (*Japonica*)	[87]
	SD	C1488, R830, R1440, R1245, C390	FC	RFLPs	5	98 BC1F5	Nipponbare (*Japonica*) × Kasalath (*indica*)	[88]
	LTG	qLTG-2, qLTG-4-1, qLTG-4-2, qLTG-5, qLTG-11	FC	RFLPs	5	98 BILs	Nipponbare (*Japonica*) × Kasalath (*indica*)	[89]
	SD	qSD-3, qSD-5, qSD-6 qSD-11	FC	RFLPs, SSRs	4	127 Double haploid (DH) lines	ZYQ8 (*indica*) × JX17 (*Japonica*)	[90]
	SD	qSD-1, qSD-3, qSD-7	FC	SSRs	3	166 F1, 12 F2,	IR50 (*indica*) × Tatsumimochi (*Japonica*) × Miyukimochi (*Japonica*) 3-way Breeding	[91]
	SD	qSdn-1, qSdnj-3, qSdn-5, qSdn-7, qSdn-11	FC	SSRs	5	168 BC1, 82 BC1, 148 F2 individuals	Nanjing35 (*Japonica*) × N22 (*indica*) and USSR (*Japonica*) × N22 (*Indica*)	[92]
	PHS	qPSR-2, qPSR-5, qPSR-8	FC	SSRs	3	164 F2	K81 × G46B	[93]
	SD	qDOR-2, qDOR-3-1, qDOR-3-2, qDOR-3-3, qDOR-5-1, qDOR-5-2, qDOR-6-1, qDOR-6-2, qDOR-8, qDOR-9-1, qDOR-9-2, qDOR-11-1, qDOR-11-2, qDOR-11-3, qDOR-11-4, qDOR-11-5, qDOR-11-6	FC	RFLPs	17	125 F7 RILs	Pei-kuh × *O. rufipogon*	[94]
	LTG	qLTG-3, qLTG-10	GC	SSRs	2	198 Double haploid F1	Zhenshan 97B (*indica*) × AAV002863 (*Japonica*)	[95]
	LTG	qLTG-3, qLTG-4, qLTG-5-1, qLTG-5-2, qLTG-5-3, qLTG-5-4, qLTG-7, qLTG-9, qLTG-10, qLTG-11-1, qLTG-11-2	GC	SSRs	11	148 F2	USSR5 (*Japonica*) × N22 (*Indica*)	[96]
	GR	qGR-2, qGR-3, qGR-11, qGR-12, qGR-2, qGR-10, qGR-11, qGR-7	GC	RFLPs, SSRs	13	71 F6 RILs	IR64 (*Indica*) × Asominori (*Japonica*)	[97]
	GI	qGI-2, qGI-7, qGI-10, qGI-11						
	MGT	qMGT-2						
	SD	qSd-1, qSd-2, qSd-1-1, qSd-1-2	GC	SSR	4	122 BILS, 536 BC6F2	N22 (*Indica*) × Nanjing35 (*Japonica*)	[98]
	SD	Sdr6, Sdr9, Sdr10	CC (Short Day)	SSRs	3	44 CSSLs, 87 F2 RILs	Koshihikari × Nona Bokra, F2 population of SL506 × Koshihikari	[99]
	LTG	qLTG-7, qLTG-9, qLTG-12, qLTG-7, qLTG-9 (Os09g0395600,Os09g0396300,Os09g0396900, Os09g0395700, and Os09g0395800)	FC	SSRs	5	F7 RILs, 181 individuals	USSR5 (*Japonica*) × N22 (*Indica*)	[100]
	SD	qSD-3.1, qSD-6.1, qSD-7.1, qSD-10.1	FC	RFLPs	4	98 BILs, 4 CSSLs	Nipponbare × Kasalath	[101]
	SD	qSD1.1, qSD2.2, qSD4.1, qSD4.2, qSD5.1, qSD2.1, qSD3.1, qSD7.1	FC	SSRs	8	150 RILs (F2:9)	Jiucaiqing (*Japonica*) × IR26 (*Indica*)	[102]
	LTG	qLTG3, qLTG7-1, qLTG7-2, qLTG12, qLTG8	FC	SSRs, STS	5	160 RILs	Tong88-7 × Millyang23	[103]
	SD	qDGE1, qDGE5a, qDGE5b, qDGE7	FC	RFLPs, SSRs	4	240 RILs	ZS97 × MH63 (Hybrid Shanyou 63)	[104]
	LTG	qLTG-3-1, qLTG-3-2, qLTG-4	FC	SSRs	3	F1 BC1F1, 122 BILs BC1F5	Hayamasari (*Japonica*) × Italica Livorno (*Japonica*)	[105]
	SD	qSDS-4, qSDS-6, qSDS-7, qSDS-8, qSDS-12	GH	SSRs	5	BC1	EM93-1 × EM93-1 (*indica*-type Breeding line) × SS18-2 (*indica* wild-type weedy rice)	[106]
	SD	qSD4, qSD7-1, qSD7-2, qSD8, qSD12	GH	SSRs	5	F1, 156 BC1	SS18-2 (weedy Rice) × EM93-1 (Cultivated rice)	[107]
	SD	qSD1-2, qSD3, qSD6, qSD7-2, qSD10	GH	SSRs	5	BC1F1	SS18-2 × EM93-1	[108]
	SD	qSD1, qSD3, qSD4, qSD7-1, qSD7-2, qSD7-3, qSD10, qSD11, qSD12	FC	SSRs	9	BR RIL 198 indivuduls, CR RIL 174 individuals F8:9 generation	s Bengal × PSRR-1; Cypresss × PSRR-1	[109]
	SD, PHS (R)	qSDR9.1, qSDR9.2	FC	SSRs	2	44 BC4F5, CSSL	Owarihatamochi × Koshihikari	[85]
	SD	qDOR-2, qDOR-3-1, qDOR-3-2, qDOR-3-3, qDOR-5-1, qDOR-5-2, qDOR-6-1, qDOR-6-2, qDOR-8, qDOR-9-1, qDOR-9-2, qDOR-11-1, qDOR-11-2, qDOR-11-3, qDOR-11-4, qDOR-11-5, qDOR-11-6	FC	RFLPs	17	189 F2, 158 F2 progency	Pei-kuh × *O. rufipogon*	[94]
	SD	qSD1-2 (* Map-based cloning)	CC	SSRs	1	BC5F3 F2 RIL	EM93-1 (*Indica*) × SS18-2 (weedy rice)	[110]
	LTG	qLTG3–1 (* Map-based cloning)	CC	SSRs, SNP, Indels	1	BILs 116	Hayamasari × Italica Livorno	[111]
	SD	Sdr4 (* Map-based cloning)	CC	FNPs, SNPs, Indels	1	28 BC4F2	Nipponbare (*Japonica*) × Kasalath (*Indica*)	[59]
	PHS	Sdr6, qSD-1, qSD1, qDEG1, qSdn-1, Sdr1, qDT-SGC3.1, qSD-3, qSdn-5, qMT-SGC5.1, Sdr9, qDOR6-2, qSD6, SDR4, qMT-SGC7.2, qSD-7-2, qPHS-7, qSD12, qLTG3-1	FC	GBS	6	21	*Japonica* (14) *Indica* (7)	[14]
GWAS	PHS	Os01g03740, Os01g03730, Os01g03820, Os01g03840, Os01g03890, Os01g03914, Os01g03950, Os04g08460, Os04g08470, Os04g08570	FC	SNPs	10	*Indica*, *Japonica*	277 accessions	[70]
	SD	RM6902, RM525, RM231, RM5963, qSD7-1, FHS7.0, RM234, FH8.1, qSD-11	FC	SNPs		*Indica*	453 accessions	[112]
	SD	GA2ox3, GH3–2, EUI1, Sdr4, GA2ox3, OsEF3, OsbohE, OsISA, OsHPL2, EXP4, OsMADS13, AP59, OsAsr1, OsABI5, OSH43, Pid3, OSH43, OsCLC-1, OsLHY, OsBOR1	FC	SNPs	20	*Indica*, *Japonica*, Aus	350 accessions	[113]

* Seed dormancy (SD), Normal Growth condition (NG), Growth chamber (GC), Green House (GH), Mean germination time (MGT), Controlled condition (CC), Simple sequence repeats (SSRs), Genotyping by sequencing (GBS), Restriction fragment length polymorphism (RFLP), Pre-harvest sprouting (PHS), Genome-wide association studies (GWASs), low-temperature germination (LTG).

**Table 2:** Application of next-generation sequencing technologies on pre-harvest sprouting in rice.

Category	Material	Plant Organ/Developmental Stage	Study Objective	Methodology	Reference
PHS	Gopum (PHS-susceptible) and Jowoon (PHS-resistant)	4 embryo and endosperm	miRNA PHS	RNASeq and small RNASeq (Illumina HiSeq 2500)	[117]
SD	Nona Bokra	Seeds (dormant and dormant broken)	SD	RNASeq (Illumina Hiseq 2000)	[116]
PHS	Gopum and Samgwang	Caryopses	PHS, SD	Microarray (Agilent DNA Microarray Scanner)	[114]
SD	Cultivar N22 and Q4646	Seeds	SD	GeneChip arrays (Affymetrix Fluidics Station 450 and GeneChip Scanner 3000)	[120]
Germination	Cultivar N22	Seedlings	GHT	RNASeq (Ion Proton sequencer)	[115]
PHS	Joun and Jopyeong	Seeds	PHS	RNASeq	[119]

Germination at high temperature (GHT); Pre-harvest sprouting (PHS); Seed dormancy (SD).

## Data Availability

All the data are shown in the article.

## Abbreviations

Abscisic acid (ABA), backcross inbred lines (BILs), chromosome segment substation lines (CSSLs), DELAY OF GERMINATION-1 (DOG-1), double haploidy (DH), genome-wide association studies (GWASs), genotyping by sequencing (GBS), germination index (GI), gibberellic acid (GA), indole-3-acetic acid (IAA), low-temperature germination (LTG), marker-assisted selection (MAS), next-generation sequencing (NGS), quantitative trait loci (QTLs), recombinant inbred lines (RILs), restriction fragment length polymorphism (RFLP), simple sequence repeats (SSRs).
